# Subhepatic appendix: an ectopic topography not to be disregarded: a case report

**DOI:** 10.1186/s13256-021-02883-6

**Published:** 2021-05-31

**Authors:** Abdoul Kadir Ibrahim Mamadou, Souleymane Mounkaila, Nouhou Hama Aghali, Mahaman Laouali Harouna Amadou, Ousseini Adakal

**Affiliations:** 1 Department of Medicine and Medical Specialties, Regional Hospital Center of Tahoua, Tahoua, Niger; 2 Department of Surgery and Surgical Specialties, Regional Hospital Center of Tahoua, Tahoua, Niger; 3 Department of Biology, Regional Hospital Center of Maradi, Faculty of Health Sciences of the University of Maradi, Maradi, Niger; 4 Department of Infectiology, Regional Hospital Center of Maradi, Faculty of Health Sciences of the University of Maradi, Maradi, Niger; 5 Department of Surgery, Regional Hospital Center of Maradi, Faculty of Health Sciences of the University of Maradi, Maradi, Niger

**Keywords:** Peritonitis, Subhepatic appendix, Embryogenesis, Case report

## Abstract

**Introduction:**

Subhepatic appendix is most often due to an anomaly of rotation of the primary intestine occurring during embryogenesis. This ectopic topography associated with advanced age can be a serious diagnosis problem at the stage of appendicitis or appendicular peritonitis.

**Case presentation:**

We report the case of a 60-year-old melanoderm man, with a history of urinary pathology and peptic ulcer, referred from a health district for abdominal pain of the right hypochondrium evolving for about 5 days, secondarily generalized, in whom we suspected peritonitis, the etiology of which remains to be determined. During the surgical intervention, after preoperative resuscitations measures, a phlegmonous perforated appendix was found under the liver. No postoperative complication was noted, and he was discharged home 8 days after his operation.

**Conclusion:**

Subhepatic appendicular peritonitis occurring in an elderly patient poses enormous diagnostic problems. When faced with right upper quadrant pain, considering acute ectopic appendicitis would significantly reduce complications.

## Introduction

Acute appendicitis, the most frequent emergency in digestive surgery, is a well-known pathology in children and young adults. Its diagnosis presents some difficulties in the elderly. Appendicitis is a source of controversy between radiologists and clinicians [1, 2]. Ectopic topographies pose serious diagnostic problems, delaying adequate surgical management. We report the clinical case of subhepatic appendicular peritonitis in a 60-year-old patient. This case is particularly interesting considering, on the one hand, the ectopic topography of the appendix, but also the age of the beginning of the pathology. Another important fact is that this article draws the attention of medical practitioners, especially in developing countries, to think about this diagnosis when faced with a similar symptomatology. This may reduce diagnostic errors, thus helping to save patients’ lives.

## Case report

We report the case of a 60-year-old melanoderm man, with a history of urinary pathology not documented 20 years ago and a peptic ulcer that was also undocumented. No significant family history was reported. He was referred from Keita Health District for abdominal pain that was first localized in the right hypochondrium before becoming generalized, with fever and vomiting, without stopping transit for about 5 days.

On admission, the patient had good general condition as well as well-colored conjunctiva and mucous membranes, with Glasgow score 15, temperature 37.3 °C, blood pressure: 120/80 mm Hg, heart rate 68 beats per minute, respiratory rate 34 cycles per minute, and oxygen saturation 99%. Physical examination revealed a soft but sensitive abdomen with a screaming umbilicus.

Proctological examination revealed a huge anal prolapse.

Laboratory investigations showed that he had a white blood cell count of 28,100/mm^3^ predominantly granulocytic; anemia at 10.7 g/dl, normocytic, normochromic; hyperplaquettosis at 528,000/mm^3^; Rhesus blood grouping O negative; normal uremia at 0.23 g/dl; normal serum creatinine at 10.13 mg/l; and normal blood sugar at 1.07 g/l.

An emergency abdominal ultrasound does not objectively detect the presence of the cecum and appendix in the iliac fossa. However, it does objectively determine the normality of the gallbladder. We do not have a computed tomography (CT) scan in the regional hospital center to further support the diagnosis.

All in all, the diagnosis of peritonitis seemed obvious, even though the etiology remains to be determined.

**Fig. 1:**
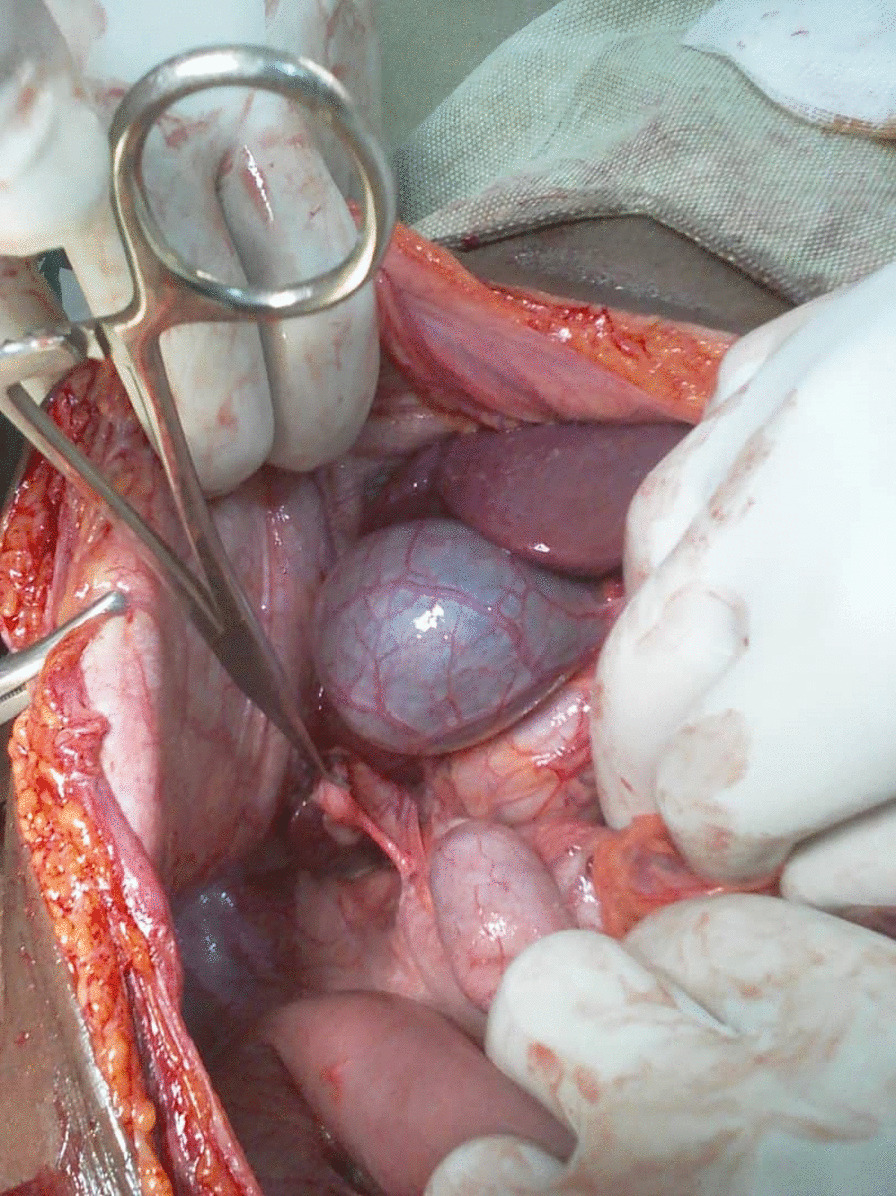
Perioperative view of subhepatic appendix

**Fig. 2:**
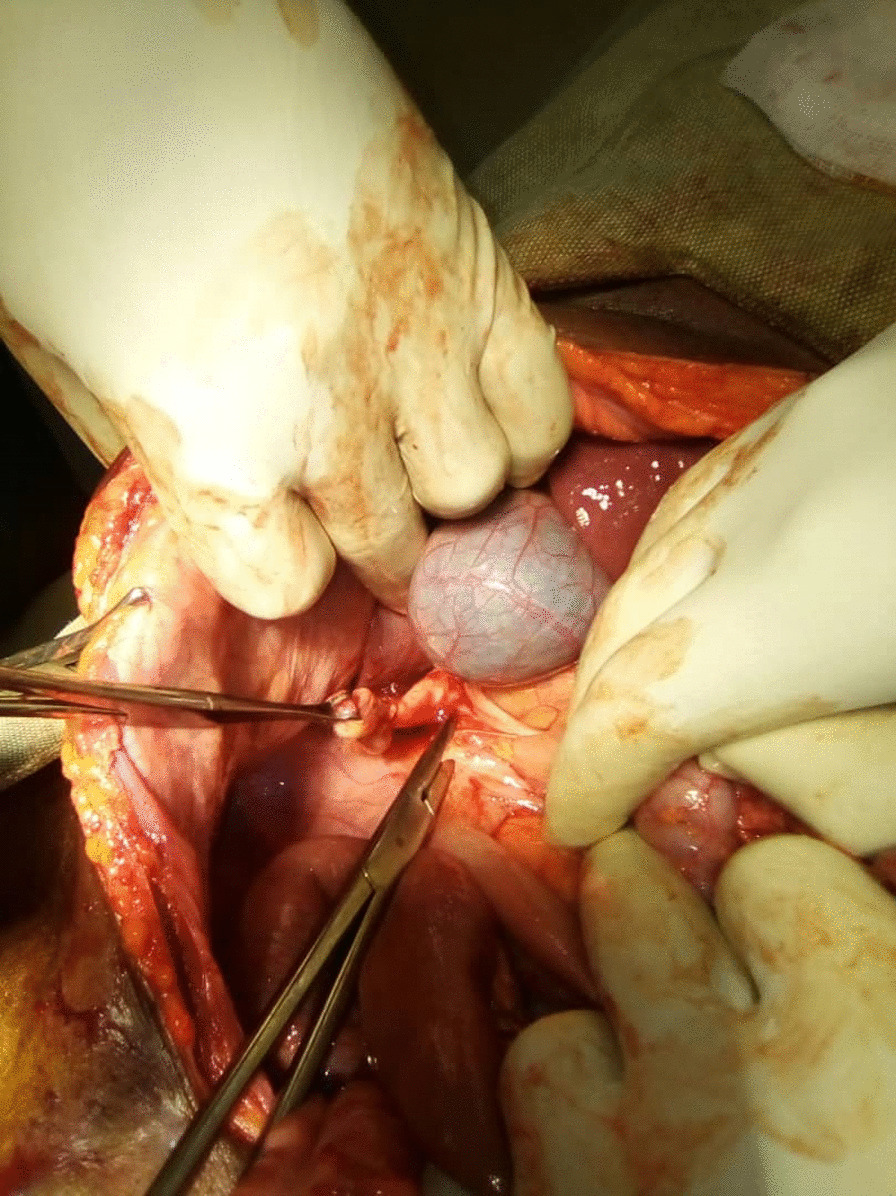
Perforated appendix under the liver

**Fig. 3:**
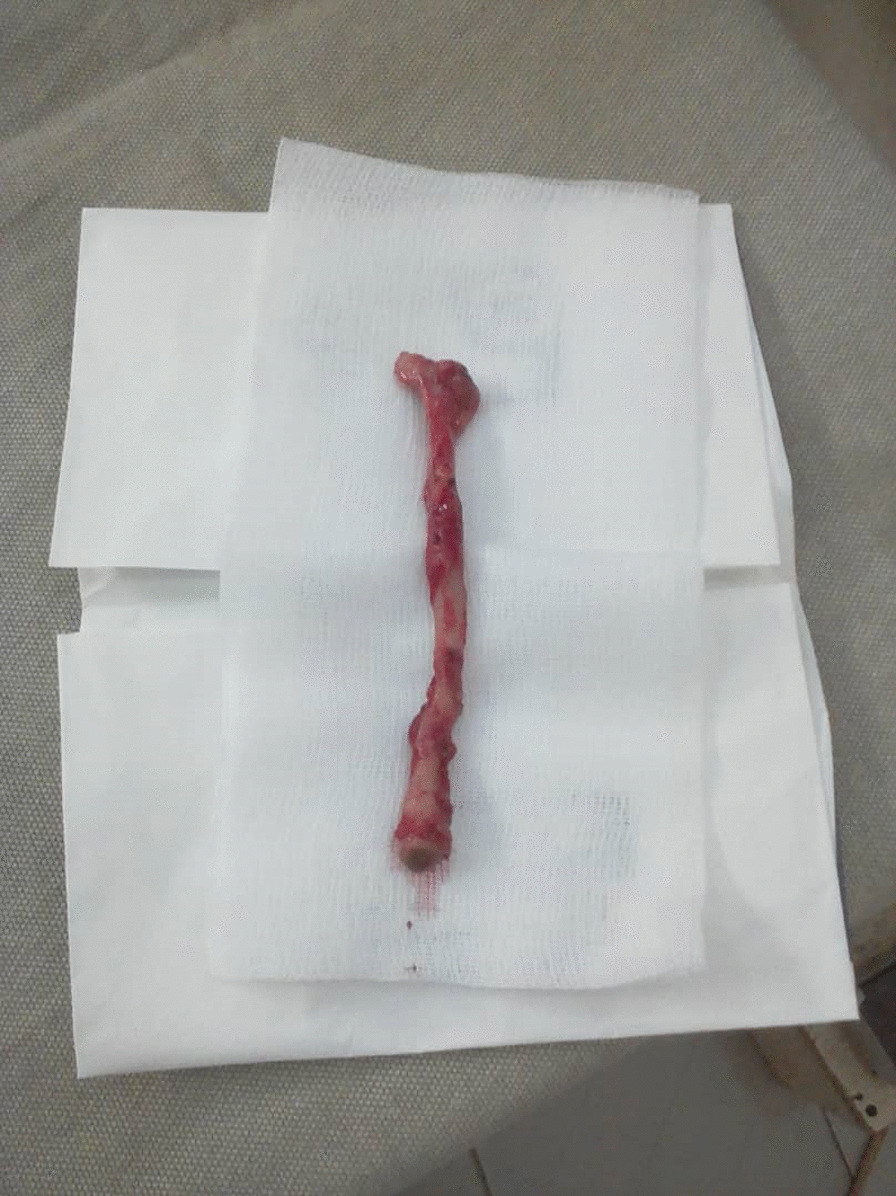
Appendix after surgical excision

After an intensive preoperative reanimation, a median laparotomy was performed. The exploration showed an acute generalized peritonitis with pus (Fig. 1), distension of the colonic frame with a left dolichocolon, false membranes, and a phlegmonous subhepatic appendix perforated. It measures up to 10 cm and is located 3 cm from the ileocecal junction (Figs. 2 and 3). An appendectomy, a hemostasis, and a promontofixation of the rectum and peritoneal toilet were subsequently performed, followed by installation of a drain in the Douglas cul-de-sac. No postoperative complication was noted, and he was discharged home 8 days after his operation with antibiotic therapy and analgesic.

## Discussion

Several works of literature admitted that “the true incidence of acute appendicitis in most developing African countries is largely unknown due to poor medical record-keeping and unreliable population census” [3].

Appendicitis is a predominantly male condition health issue, and it occurs more often in young people than in the elderly. Elsewhere, there has been a significant seasonal effect, with an increased frequency in summer [4, 5].

The location of the appendix in the subhepatic position has two origins—an exceptional one called subhepatic appendix, of which few cases have been reported in the literature so far, and a more common one corresponding to a retrocecal appendix ascended below the liver. The exception is due to a rotation abnormality of the primary intestine occurring during the 11th week of embryonic development and placing the cecum in the upper quadrant [6].

Diagnosis of appendicitis has relatively low accuracy in the elderly. Despite the infrequent occurrence of appendicitis, the rate of perforation is always unfavorable. Postoperative morbidity and mortality are too high. Advanced age adversely affects the clinical diagnosis, the stage of the disease, and patient outcomes. Perforated appendicitis and septic progression are the main cause of poor outcomes [7, 8].

The subhepatic position of the appendix results in unusual, nonspecific clinical symptoms, delaying diagnosis to the stage of appendicular rupture [6]. There are many positional variations of the appendix in relation to the cecum: mediocecal, the most frequent, retrocecal, in contact with the right iliac psoas muscle, anterior or posterior subcecal, prececal, and anterior or posterior ileocecal. Similarly, positional variations of the cecum or length of the appendix will determine subhepatic, pre- or retrocolonic appendicitis and pelvic appendicitis [9].

An x-ray of the abdomen without preparation is an insufficient or even useless examination in the diagnosis of acute appendicitis. Ultrasound is often sufficient for a positive diagnosis of acute appendicitis, especially in young subjects. Computed tomography scan is necessary and indispensable in complicated forms (especially appendicular perforations) and for most ectopic forms [9, 10].

A meta-analysis suggests that although antibiotics may be used as primary treatment for some patients suspected of having uncomplicated appendicitis, they are unlikely to supplant appendectomy at this time. Selection bias and the shift to surgery in the three randomized controlled trials suggest that appendectomy is the gold standard therapy for acute appendicitis [11].

Acute appendicitis in the elderly is always associated with significant morbidity. Preexisting severe comorbidities are a major contributing factor to mortality in these patients. The morbidity in one series was exclusively parietal suppuration [1, 12].

In developing countries, due to limited means of investigation and precarious health facilities, similar pathologies can go unnoticed, unfortunately affecting the vital prognosis. It is imperative to equip our centers with high-performance imaging resources to make these types of diagnoses at the preoperative stage.

## Conclusion

Ectopic appendicular peritonitis in old age poses enormous diagnostic difficulties. A rapid positive diagnosis combined with appropriate management would reduce the high morbidity and mortality associated with this condition.

## Data Availability

Data sharing not applicable to this article as no datasets were generated or analyzed during the current study.
